# Compact Electron Paramagnetic Resonance on a Chip Spectrometer Using a Single Sided Permanent Magnet

**DOI:** 10.1021/acssensors.4c00788

**Published:** 2024-09-26

**Authors:** Michele Segantini, Gianluca Marcozzi, Tarek Elrifai, Ekaterina Shabratova, Katja Höflich, Mihaela Deaconeasa, Volker Niemann, Rainer Pietig, Joseph E. McPeak, Jens Anders, Boris Naydenov, Klaus Lips

**Affiliations:** † Helmholtz-Zentrum Berlin für Materialien und Energie GmbH, Hahn-Meitner-Platz 1, 14109 Berlin, Germany; ‡ Institute of Smart Sensors, Universität Stuttgart, 70569 Stuttgart, Germany; § Leibniz-Institut für Höchstfrequenztechnik, Ferdinand-Braun-Institut gGmbH, 12489 Berlin, Germany; ∥ Bruker BioSpin GmbH, 76275 Ettlingen, Germany; ⊥ Center for Integrated Quantum Science and Technology, 70569 Stuttgart and Ulm, Germany; # Berlin Joint EPR Laboratory, Fachbereich Physik, Freie Universität Berlin, 14195 Berlin, Germany

**Keywords:** EPR, EPRoC, single side permanent magnet, spin counting, in situ, in vivo, operando, molecular tumbling

## Abstract

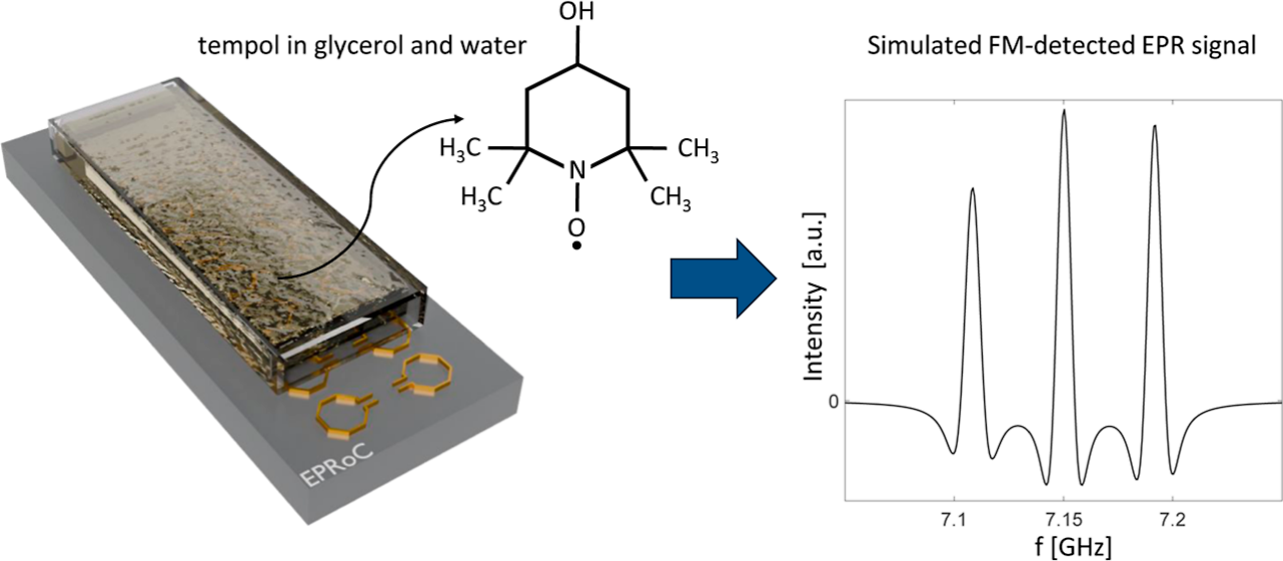

Electron paramagnetic resonance (EPR) spectroscopy provides information about the physical and chemical properties of materials by detecting paramagnetic states. Conventional EPR measurements are performed in high *Q* resonator using large electromagnets which limits the available space for operando experiments. Here we present a solution toward a portable EPR sensor based on the combination of the EPR-on-a-Chip (EPRoC) and a single-sided permanent magnet. This device can be placed directly into the sample environment (i.e., catalytic reaction vessels, ultrahigh vacuum deposition chambers, aqueous environments, etc.) to conduct in situ and operando measurements. The EPRoC reported herein is comprised of an array of 14 voltage-controlled oscillator (VCO) coils oscillating at 7 GHz. By using a single grain of crystalline BDPA, EPR measurements at different positions of the magnet with respect to the VCO array were performed. It was possible to create a 2D spatial map of a 1.5 mm × 5 mm region of the magnetic field with 50 μm resolution. This allowed for the determination of the magnetic field intensity and homogeneity, which are found to be 254.69 mT and 700 ppm, respectively. The magnetic field was mapped also along the vertical direction using a thin film a-Si layer. The EPRoC and permanent magnet were combined to form a miniaturized EPR spectrometer to perform experiments on tempol (4-hydroxy-2,2,6,6-teramethylpiperidin-1-oxyl) dissolved in an 80% glycerol and 20% water solution. It was possible to determine the molecular tumbling correlation time and to establish a calibration procedure to quantify the number of spins within the sample.

Electron paramagnetic resonance (EPR) is a spectroscopic technique that detects paramagnetic species and free radicals, allowing the quantification and identification of physical, chemical, and biological substances. EPR spectroscopy is highly sensitive, quantitative, noninvasive and enables the analysis of various samples without complex preparation. Among other applications, it is possible by means of EPR to probe the free radicals in the human body for quantification of reactive oxygen species,^1−4^ in edible oil to monitor oxidative stability,^5−8^ in beer to ensure flavor stability,^9−12^ to detect defects in semiconductors^13−15^ and to measure the concentration of paramagnetic centers in vanadium redox flow batteries for monitoring the state of charge and health of the battery.^16,17^ Most commercially available EPR devices are bulky and power-hungry due to the presence of electromagnets that produce the external magnetic field *B*_0_. A typical EPR experiment requires the use of microwave sources and detectors in combination with high-quality factor (*Q*) microwave resonators (active volume ∼250 mm^3^ at 9 GHz), where the sample is placed inside. Due to the large *Q*-factor, microwave resonators typically have limited bandwidth, therefore the electromagnets are used to sweep the B_0_ field, while keeping the microwave frequency constant to measure the EPR spectrum. Even though EPR has been proven to be a useful tool for some in vivo, in situ and operando experiments, for example to study pO_2_ distribution,^3,4^ lithium-ion batteries,^18,19^ catalyst materials^20−23^ and for imaging,^24−26^ the implementation in conventional EPR spectrometers is challenging and limit these application. In most cases, samples must be properly designed to respect the geometrical constraints of the microwave resonator.^18,20,27^ Moreover, polar solvents must be avoided due to their large dielectric constant, which would lower the *Q*-factor of the resonator. A promising solution to overcome these limitations is provided by EPR-on-a-chip (EPRoC) technology, where the entire apparatus for EPR sensing is reduced to a small, portable, and versatile device that can be directly installed in the sample environment.^28,29^ The EPRoC is comprised of an array of voltage-controlled oscillator (VCO) coils that oscillate at a specific microwave frequency, allowing the replacement of the resonator with the VCO array for coupling of microwaves into the sample environment while maintaining a high apparent *Q*-factor.^15,30,31^ Moreover, due to the possibility of sweeping the frequency of the VCO array to perform EPR measurements,^32,33^ the portability of the EPRoC is further enhanced when used in combination with a permanent magnet.^34^

In this work, a small single-sided permanent magnet has been developed with the aim to produce a static magnetic field B_0_ of approximately 250 mT to satisfy the resonance condition for small organic radicals (*g* ≈ 2) using a VCO array oscillation frequency of ∼7 GHz. The goal of designing this permanent magnet was to minimize its size and weight to ensure high versatility and allow the installation together with the EPRoC in complex sample environments, such as in ultrahigh vacuum (UHV) evaporation chambers and catalytic reactors.^35^ The VCO array produces the oscillating out-of-plane magnetic B_1_ field and was designed employing a 0.13 μm BiCMOS technology with 14 coils.^36^ The number of coils was extended compared to previous works^37^ to improve the signal-to-noise ratio (SNR)^35^ of the EPR measurements, achieving spin sensitivity of , as reported in ref (36). In this report, a single coil of the VCO array was used as a sensor to create a 2D image of the magnetic field intensity of the permanent magnet. These results highlight the capabilities of the EPRoC to fully determine the intensity and the homogeneity of the magnetic field with high spatial resolution (50 μm), demonstrating a 10-fold improvement in the spatial resolution compared to previous reports.^26^ In addition, the whole array of the EPRoC was used to map the magnetic field along the vertical direction. The EPRoC and the permanent magnet were then combined to measure tempol (4-hydroxy-2,2,6,6-teramethylpiperidin-1-oxyl) dissolved in a mixture of glycerol and water with the aim to evaluate the possibility of performing quantitative measurements. As expected theoretically, the results of this investigation show a linear dependence of the signal intensity from the sample concentration, as confirmed by a commercial benchtop EPR system, allowing the calibration of the EPRoC for spin quantification. Finally, the tumbling correlation time of tempol in solution was determined with the EPRoC and the permanent magnet and compared to the commercial benchtop EPR spectrometer. This experiment was conducted to evaluate the capability of using the EPRoC for molecular tumbling analysis and viscosity determination.^38,39^

Overall, these experiments using the EPRoC technology serve as a proof-of-concept to open new pathways to applications that are not accessible with conventional EPR spectrometers. Among these applications, the EPRoC can be used as an alternative solution to the commonly used magnetic field cameras based on magnetometry^40^ and to design and test devices for magnetic cell manipulation.^41^ Moreover, EPRoC can be implemented directly into the sample environment and used as a “dipstick” sensor^42^ or as mobile surface explorer^43^ to determine the number of spins and to measure the molecular tumbling rate of spin labels for in vivo, in situ and operando EPR experiments.

## Materials and Methods

### Permanent Magnet

**Figure 1:**
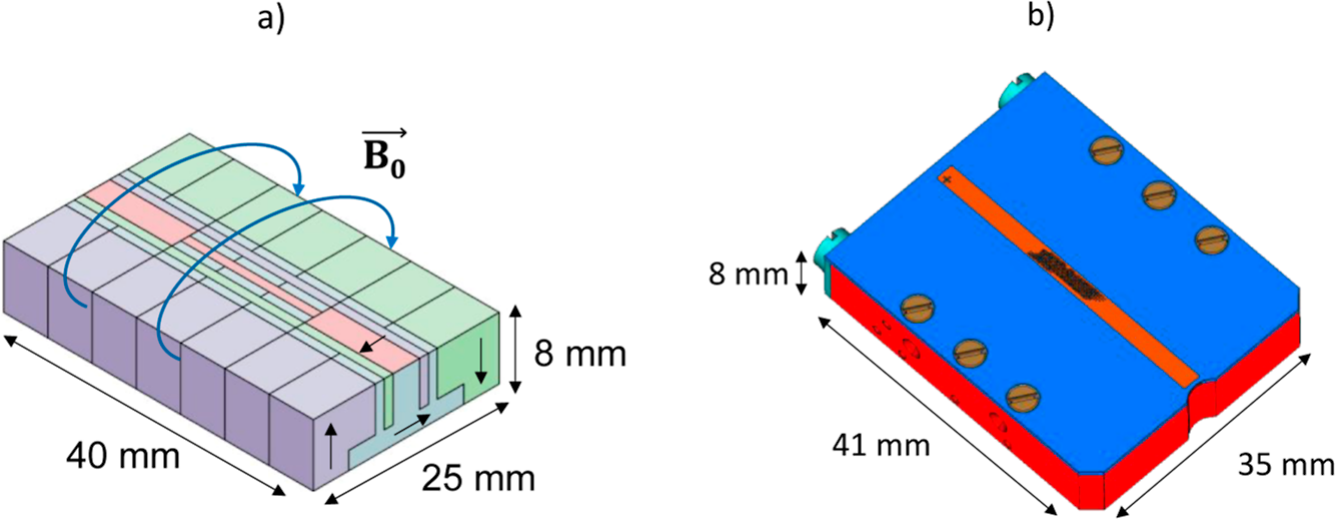
(a) The permanent magnet was assembled by gluing together small segments of samarium–cobalt, shown here with different colors highlighting their orientation, to generate a homogeneous magnetic field B0 parallel to the surface of the magnet. The dimensions of the magnet are shown (40 mm × 25 mm × 8 mm). (b) A schematic of the aluminum cage used to provide structural support for the permanent magnet. On the top of the central region of the magnet, an etched μ-metal foil (orange) of approximately 1 mm × 40 mm × 0.05 mm is placed to improve the magnetic field homogeneity. The black pattern on top of the magnet corresponds to the etched region of the μ-metal. The final dimensions of the assembled magnet are shown (41 mm × 35 mm × 8 mm).

A small single-sided permanent magnet has been designed to be combined with the 7 GHz EPRoC array. The decision to design a single-sided permanent magnet for use with the EPRoC was driven primarily by the need for enhanced accessibility and integration for in situ experiments. This geometry allows for a more flexible access to the sample area, which is crucial for the integration of the EPRoC and permanent magnet in various experimental apparatuses, enhancing its applicability in a broad range of in situ studies where conventional multisided magnets would be too bulky or restrictive. The magnet geometry was determined to respect the design criteria of a magnetic field intensity of 250 mT with a homogeneous region (≈100 ppm) of approximately 8 mm × 1 mm × 0.5 mm above the central area in the x, y and *z* directions, respectively. The magnetic materials (samarium–cobalt) were selected to ensure temperature stability of the magnetic field in the range of 20–150 °C and compatibility with UHV to allow the installation in UHV deposition chambers. Simulations of the magnet geometry were performed by combining analytical solutions of the block-shaped magnet segments with uniform magnetization (Wolfram Mathematica) and Finite Element Analysis (FEM—Ansys Maxwell). Figure 1a shows a schematic of the permanent magnet, which was fabricated by gluing (Delo MONOPOX AD295) together 32 small segments (from 27 to 364 mm^3^) of samarium–cobalt. Each segment was oriented in such a way as to generate a magnetic field *B*_0_ parallel to the surface of the magnet. This orientation was required to fulfill the orthogonality condition between the B_0_ field and the microwave magnetic field B_1_ generated by the EPRoC to perform EPR measurements. The final dimensions of the assembled magnet are 40 mm × 25 mm × 8 mm. The magnet was covered (Figure 1b) with an aluminum cage to provide additional support and to increase rigidity of the assembled structure to better resist mechanical stress. After the fabrication process, the magnetic field was measured using a Hall-probe sensor (3-axis Hall Probe C—Senis AG) and a Teslameter (3MH6 High-Precision, Low-Noise Teslameter—Senis AG) and the homogeneity estimated as the difference of the observed maximum and minimum values of magnetic field intensity, was found to be approximately 10,000 ppm. To further improve the homogeneity, a μ-metal strip was placed in the central region on the top of the magnet (Figure 1b) to locally weaken the magnetic field and thereby increase the overall homogeneity. The pattern of the μ-strip was etched using a laser (Laser StarFiber 150 P SM from COHERENT). The laser-etched pattern in the μ-metal foil allows for precise control of flux distribution enhancing the uniformity of the magnetic field and improving the overall field homogeneity in the region of interest. After placement of the μ-metal foil, the obtained homogeneity was approximately 1000 ppm. The UHV compatibility of the permanent magnet was examined by placing the permanent magnet in an UHV chamber, in which values of pressure below 7 × 10^–9^mBar have been achieved.

### Samples

Three different types of samples were used for the experiments. A single grain of α,γ-bisdiphenylene-β-phenylallyl (BDPA complex, 1:1 with benzene, purchased from Sigma-Aldrich) was used as a point-like sample to perform the 2D mapping of the static magnetic field B_0_ of the permanent magnet. The diameter of the sample (≈200 μm) was estimated from an image of the sample placed on a single coil recorded using an optical microscope (Motic, SMZ168 Series). A 15 μm thin film unhydrogenated amorphous silicon (a-Si) sample deposited using electron-beam evaporation (emission current: 560 mA, *T* = 680 °C, deposition rate ∼400–450 nm/min, sample rotation 15 rpm) from a solid source of undoped polycrystalline silicon on a 5 cm × 5 cm × 500 μm quartz substrate was used as a quasi-two-dimensional sample to cover the entire VCO array to determine the strength of the B_0_ field while increasing the distance between the array and the permanent magnet. The paramagnetic silicon dangling bonds defects are homogeneous distributed within the 15 μm thin a-Si film. The sample was then cut into small pieces (1 mm × 2.5 mm) in order to fit on the array sensor using a dicing saw (DISCO DAD3220). Four samples with different tempol (4-hydroxy-2,2,6,6-teramethylpiperidin-1-oxyl) concentrations (63, 31.7, 15.8 and 7.9 mM) dissolved in a solution of 80% glycerol and 20% water were also used for the experiments. The tempol solutions were placed in thin flat capillaries (50 μm × 1 mm × 2 mm), which were sealed with a vinyl plastic compound (Critoseal). The wall thickness of the flat capillaries was 50 μm.

### Experimental Configuration

**Figure 2:**
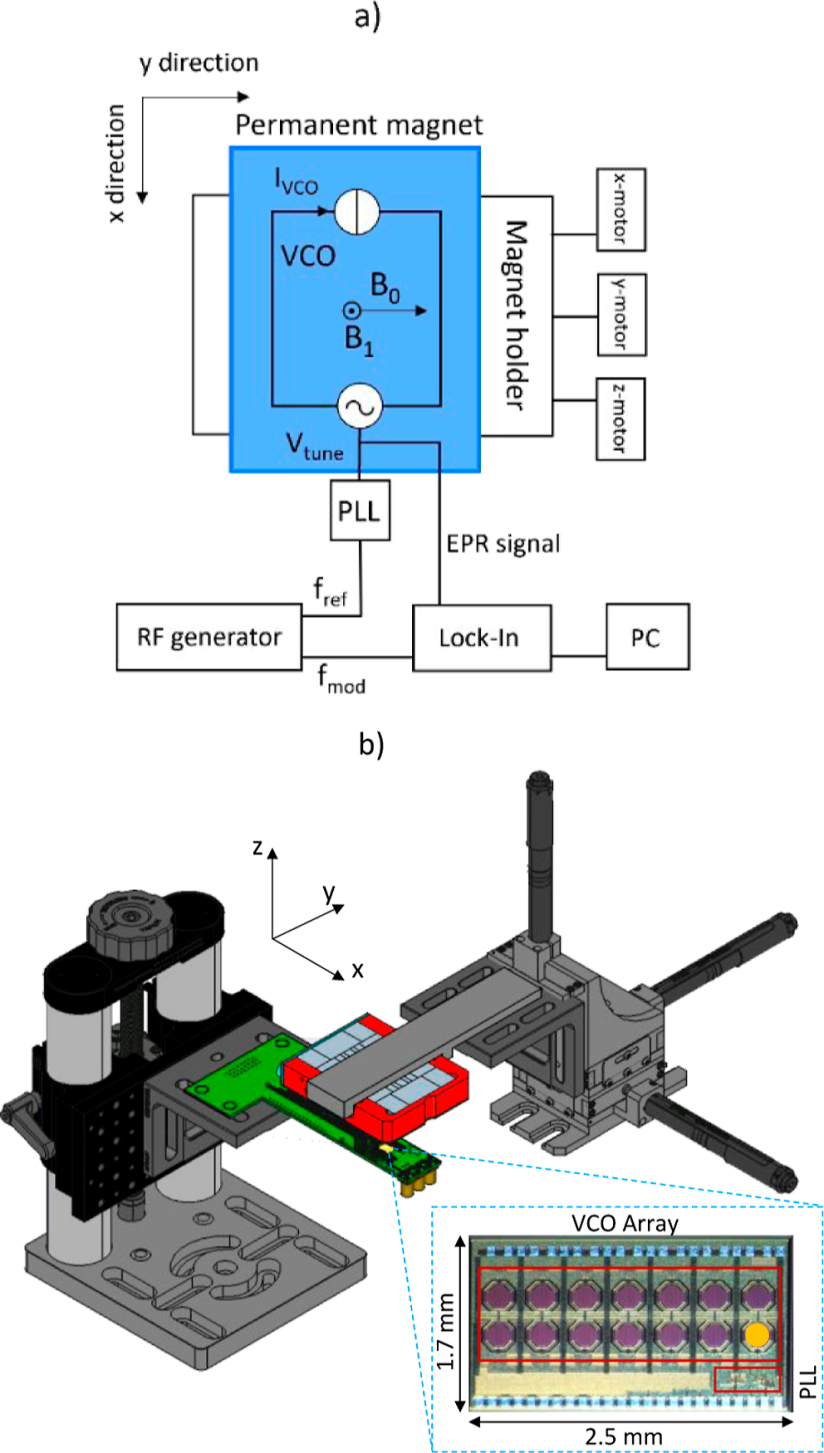
(a) Block diagram of the EPRoC and necessary components. The RF generator supplies the reference frequency *f*_ref_ to the PLL integrated on the EPRoC. The current in the VCO array coils *I*_VCO_ generates the *B*_1_ field. *V*_tune_ is the tuning voltage. When the sample goes into resonance, the oscillation frequency of the VCO changes along with the Vtune, which is measured by a lock-in amplifier to record the EPR signal. The modulation frequency *f*_mod_ is supplied from the RF generator to the lock-in amplifier. The permanent magnet (blue) is placed above the EPRoC. (b) 3D model of the scanning EPRoC setup with the permanent magnet. The PCB (green) with the VCO array (yellow) is mounted on a translation stage that can be moved along the vertical direction. The magnet is positioned above the PCB and is held by a 3D printed mounting bracket, which is connected to three DC servo motors which are used to control position in the *x*-, *y*-, and *z*-direction with respect to the EPRoC. In the rectangular dashed box, an enlarged view of the 14-coil EPRoC array is shown. The orange circle represents the BDPA grain that was placed on the coil for the magnetic field mapping. The PLL integrated on the EPRoC is indicated.

The EPRoC sensor used in this work was based on an LC VCO array designed to oscillate at 7 GHz and fabricated using a 0.13 μm BiCMOS technology.^36^ Compared to previous works,^37^ the number of coils was extended to 14, as the signal-to-noise ratio (SNR) increases , as reported in.^35,37^ The estimated spin sensitivity of the array is .^36^ The EPRoC was bonded onto a single printed circuit board (PCB), which was then placed below the permanent magnet. The experimental configuration is comprised of the translation elements required to calibrate the position of the permanent magnet and the instruments necessary to perform EPR measurements using the EPRoC. In Figure 2a the block diagram of the experimental configuration is shown. The reference frequency *f*_ref_ for the EPR measurement was supplied to the EPRoC using a signal generator (Rohde & Schwarz SMB100B). The electrical power was supplied by a voltage source (Keysight E3646A) and the EPR signal was recorded using a lock-in amplifier by monitoring the variation of the *V*_tune_ (Anfatec eLockIn 203). The *f*_ref_ defines the oscillation frequency *f*_VCO_ of the VCO array according to the relation *f*_VCO_ = 4 × *f*_ref_, where the factor 4 is given by the frequency divider integrated into the EPRoC. The current *I*_VCO_ flowing through the VCO produces the microwave *B*_1_ field, which is used to bidirectionally excite and detect the electron spins. The variation of the magnetization of the sample is detected as EPR signal by monitoring the variation of the oscillation frequency of the VCO. In this way, it is possible to sweep the VCO frequency to detect the EPR signal by recording the tuning voltage *V*_tune_ of the phase-locked loop (PLL). The VCO frequency can be sinusoidally modulated to detect the EPR signal
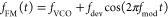
1where the *f*_FM_ is the modulated microwave frequency, *f*_dev_ is the frequency deviation, and *f*_mod_ is the modulation frequency, which is used as an external reference frequency for the lock-in amplifier. The frequency swept FM-detected EPR signal yields a dispersion-like derivative line shape.^34^Figure 2b shows a 3D model of the experimental realization of the scanning setup that was used to map the magnetic field homogeneity of the permanent magnet. The PCB, shown in green, has a T shape where the shorter side is 40 mm in length and the longer side is 78 mm in length. All electronic components necessary to operate the EPRoC, which is placed on the top side of the PCB and is shown in yellow, are placed on the back side of the PCB. This allowed the possibility of placing the permanent magnet in closer proximity to the EPRoC to perform the EPR measurements for imaging the magnetic field. The position of the magnet was varied by way of three DC servo motors (KDC101 ThorLabs) which are controlled with 1 μm precision over a maximum distance of 25 mm. The PCB was mounted on a long-traveling range translation stage (VAP10/M ThorLabs), which allowed for movement of the EPRoC along the vertical direction. The permanent magnet was fixed on a 3D printed mounting bracket fastened to three motorized translation stages (MT1/M-Z8 ThorLabs) to allow for movement of the magnet with respect to the EPRoC in three spatial directions (x, y, z). All necessary components were placed on an optical table to ensure mechanical stability during the measurements. To obtain reference spectra, frequency swept EPRoC measurements were performed by inserting the EPRoC and the PCB between the poles of an electromagnet a Bruker EPR300 X-band spectrometer.^32^ An enlarged view of the VCO array with 14 octagonal coils is shown in the inset. Depending on the experiment, the sample can be placed either on a single coil (orange circle) or onto the entire array. The PLL is embedded in the EPRoC, whose architecture is similar to the one reported in.^35^

### EPRoC Measurements

The EPR measurements of the three samples were performed using the EPRoC in the frequency sweep mode.^44^ A single grain of BDPA was placed on a single coil of the VCO array to map the magnetic field of the permanent magnet. The sample was first measured in the electromagnet to determine the line width observed when the magnet homogeneity is ≈ 40 ppm. As it has been reported in previous works, the EPRoC used in the frequency sweep mode has a baseline drift induced by the conversion of the tuning voltage to the oscillation frequency of the VCO.^44^ The EPR signal is recorded by monitoring *V*_tune_, using a lock-in amplifier, and the baseline drift can be described by an arbitrary polynomial function (approximate fourth order). Therefore, to perform the baseline correction, an “on-resonance” spectrum was recorded by fixing the magnetic field intensity at *B*_0_ = 255.7 mT. An “off-resonance” spectrum, measured at *B*_0_ = 260.0 mT, was then subtracted from the on-resonance spectrum. These measurements were performed using frequency modulation *f*_mod_ = 100 kHz, frequency deviation *f*_dev_ = 0.4 MHz, which allowed for integration of the detected signal over a bandwidth equivalent to ≈0.014 mT magnetic field modulation in a typical EPR experiment, time constant of the lock-in amplifier τ = 20 ms, total frequency sweep width of 48 MHz and *N* = 1201 data points. The BDPA grain was then measured with the permanent magnet using the same experimental parameters; however, only *N* = 534 data points were recorded. In this case, the off-resonance spectrum required for the baseline correction was obtained by moving the permanent magnet 2 cm away from the EPRoC. In this configuration, the EPRoC was no longer under the permanent magnet, and thus outside the region of the *B*_0_ field. The 2D mapping procedure was performed by translating the magnet along the *x*- and *y*-direction using steps of 50 and 100 μm, respectively, keeping the distance between the EPRoC and the permanent magnet fixed at 300 μm. The measurements on the thin film a-Si sample were recorded as the average of 20 spectra for each distance using frequency modulation *f*_mod_ = 100 kHz, frequency deviation *f*_dev_ = 5.2 MHz, time constant of the lock-in amplifier τ = 20 ms, total frequency sweep width 137.2 MHz and *N* = 502 data points. The magnetic field strength along the vertical direction was determined by moving the position of the magnet in steps of 50 μm along the *z*-direction. The measurements of tempol in 80% glycerol and 20% water solution were recorded as the average of 30 spectra using frequency modulation *f*_mod_ = 100 kHz, frequency deviation *f*_dev_ = 1.3 MHz, time constant of the lock-in amplifier τ = 20 ms, total frequency sweep 204 MHz and *N* = 511 data points. The four tempol solutions with 63, 31.7, 15.8 and 7.9 mM dissolved in 80% glycerol and 20% water were also measured using a Magnettech MS5000 spectrometer with 100 kHz modulation frequency, *B*_1_ = 3.8 μT, and 40 s of sweep time. The maximum value of *B*_1_ = 20 μT at the sample position 50 μm above the EPRoC surface was estimated from the Biot-Savart law according to the procedure showed in.^45^ The tempol spectra measured with the EPRoC and permanent magnet were filtered using a second order Savitzky–Golay filter with a window of 6 data points in order to prevent line shape broadening beyond 5%. For controlling the experiments and the data acquisition with the EPRoC and the permanent magnet, a customized version of the software package Qudi was used.^46^ Simulations were performed using the pepper function of the EasySpin library in MATLAB (Mathworks) for simulating power-averaged spectra of the thin-film a-Si sample to obtain a reference spectrum for the calculation of the line width broadening induced by the permanent magnet.^47^ The a-Si spectrum was simulated using the values of the *g*-factor and line width (0.38 mT) at 7.3 GHz reported in literature.^13,45^ The baseline correction for the double integration procedure was performed using the backcor^48^ function in MATLAB for a first order polynomial utilizing an asymmetric truncated quadratic function and values of the threshold that were chosen to avoid signal distortions. The least-squares fitting routine using the esfit function of the EasySpin library in MATLAB was performed to determine the line width and the correlation time of the 31.7 mM tempol solution in 80% Glycerol and 20% water.^47^ Due to the high viscosity of the solution, the chili function for simulating the slow-motion regime was used in the fitting procedure. Moreover, a field offset parameter to compensate for the uncertainty of the magnetic field due to the positioning of the permanent magnet with respect to the EPRoC was included in the least-squares analysis.

## Results and Discussion

### 2D Magnetic Field Mapping

**Figure 3:**
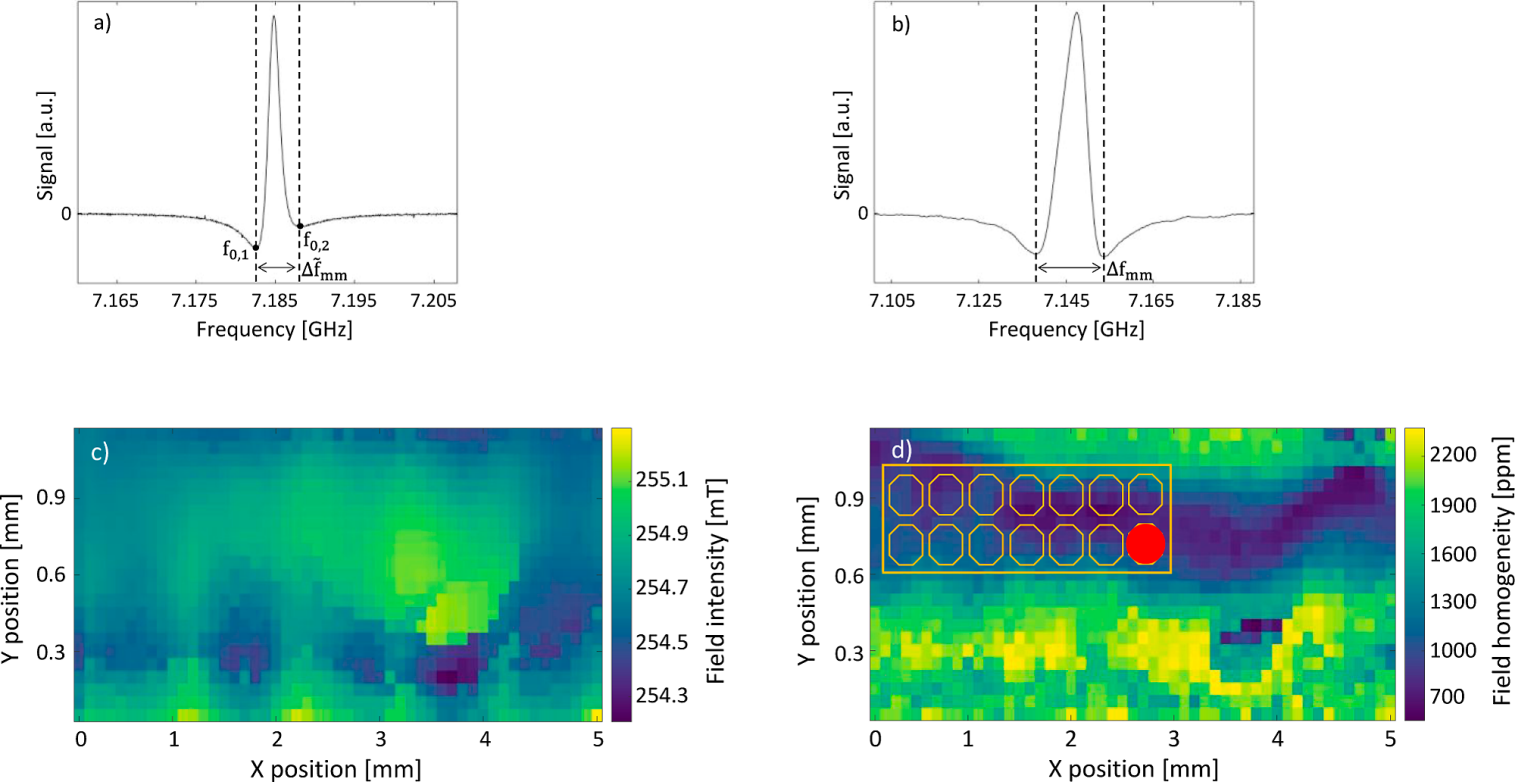
(a) FM-detected EPR spectrum of BDPA measured using the 7 GHz EPRoC array in an electromagnet with high homogeneity (≈40 ppm). The distance  between the two minima is used as a reference value. (b) EPR spectrum of BDPA measured using the 7 GHz EPRoC array and the permanent magnet. The distance Δ***f***_**mm**_ = 15.5 MHz between the two minima is measured for each EPR spectrum during the mapping procedure. It should be noticed that in Figure 2a the resonance occurred at **B**_0_ = 255.7 mT, which is ≈1 mT higher than the average magnetic field in the homogeneous region of the permanent magnet. This discrepancy is due to the magnetic field offset occurring when positioning the EPRoC between the poles of the electromagnet. (c) 2D mapping of the intensity of the magnetic field of the permanent magnet. The map is obtained via calculation of the resonance position of the EPR spectrum at each spatial position. The pixel size is 50 μm × 100 μm (*x*, *y*), and the total mapped area is 5 mm × 1.15 mm. (d) 2D map of the broadening (in ppm) of the EPR spectral line induced by the inhomogeneity of the magnetic field of permanent magnet. The orange dashed rectangle containing 14 octagonal shapes depicts (to scale) the EPRoC sensors and where the red dot represents the BDPA grain used to perform the mapping procedure.

The EPRoC has been used as a sensor to determine the intensity and homogeneity of the magnetic field *B*_0_ of the permanent magnet by recording the EPR spectra of a point-like BDPA sample placed on a single coil of the 7 GHz array. All measurements were performed by sweeping the frequency of the EPRoC. The first measurements were carried out using the EPRoC and the conventional electromagnet to characterize the properties of the EPRoC spectrum of BDPA. The spectrum shown in Figure 3a was recorded using the electromagnet, and the distance between the two minima () of the FM-detected signal was used as reference value for the line width of the spectrum which was obtained from the total frequency deviation. Figure 3b shows the result of the measurements obtained using the permanent magnet. The position of the resonance peak and the distance between the two minima (Δ*f*_mm_ ≈ 12.6 MHz) were used to determine the value of the field (*B*_0_ = 254.95 mT) and its homogeneity by comparing with the reported BDPA *g* value, 2.0026, and the increase in line width from the recorded reference spectrum shown in Figure 3a, respectively.^49^Figure 3c,d show the 2D map of the intensity and the homogeneity of the magnetic field at the position of the EPRoC sensor above the permanent magnet, respectively. The mapping was performed by translating the permanent magnet with respect to the BDPA grain placed on one of the EPRoC sensors while measuring the EPR spectrum at each position with a step size of 0.1 mm. The distance between the EPRoC and the magnet was *d* = 300 μm and was not varied throughout the mapping procedure. The intensity of the *B*_0_ field was determined at each spatial position from the resonance position of the EPR spectrum of the BDPA. The value of the magnetic field over the entire mapped area of the permanent magnet lies within the interval *B*_0_∈[253.22, 256.45], with the weighted average value . The distance between the two minima of each spectrum Δ*f*_mm_ was used to determine the signal broadening, Δ*B*_ppm_ in ppm, induced by the inhomogeneity of the field using the following relation
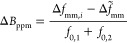
2where Δ*f*_mm,*i*_ is the distance between the two minima of the FM-signal measured with the permanent magnet at the *i*th-position,  is the reference value of the distance between the two minima of the FM-signal measured using the electromagnet, and *f*_0,1_ and *f*_0,2_ are the corresponding frequency values of the two minima in the reference spectrum, see Figure 3a. The full derivation of the eq 2 can be found in the “Broadening derivation” Section in the Supporting Information. The results of these measurements demonstrate that the highest homogeneity is ≈700 ppm, as shown in Figure 3d, blue, and it stretches over about 40% of the investigated region. This value is comparable with the value obtained from the Hall probe measurements. It should be noted that the absolute field intensity using the EPRoC sensor directly as a Hall probe like device, shown in Figure 3c, where the 2D map directly describes the magnetic field, provides a sufficient measurement of homogeneity. However, the line width of the selected EPR sample provides additional information regarding the effects of homogeneity on the accuracy (in ppm) of the intended sensing application as it directly indicates the limitations of measuring the EPR signal with the EPRoC device.

### Magnetic Field Mapping along the Vertical Direction

**Figure 4:**
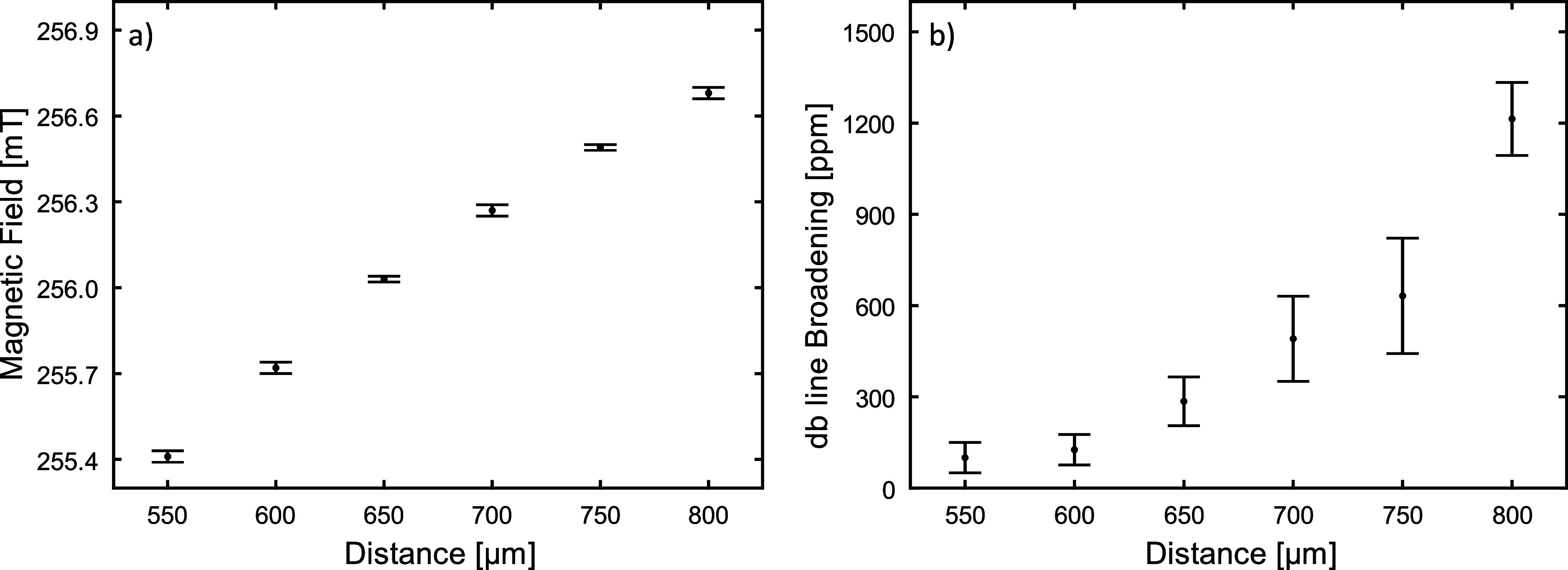
(a) Dependence of the *B*_0_ intensity as a function of the distance between the EPRoC array and the permanent magnet. The data were obtained via calculation of the db resonance position of the a-Si sample placed on the entire VCO array. (b) Dependence of the dangling bonds signal broadening measured in ppm as a function of distance between the EPRoC array and the permanent magnet. The errors are indicated at each data point.

We also evaluated the magnetic field intensity and homogeneity along the vertical direction of the permanent magnet. In this case the FM signal of the neutral dangling bonds (db), which are homogeneously dispersed in a 15 μm thin film of amorphous silicon (a-Si), was measured. Due to its dimensions, the a-Si sample covered the entire VCO array to achieve uniformly distributed db spins on each coil of the VCO. In this way, it was possible to determine the line width broadening induced by the magnetic field inhomogeneity when all coils of the array are active. Based on the results of the 2D mapping, the *x*- and *y*-positions of the EPRoC with respect to the permanent magnet were selected such that the EPRoC would be placed in the region of highest homogeneity thereby minimizing signal broadening. The distance between the EPRoC and the magnet was increased in steps of 50 μm, and the EPR spectrum was recorded at each position. The intensity of B_0_ and the broadening of the EPR signal were determined via calculation of the resonance position using the characteristics of the a-Si db signal at 7.3 GHz (*g* = 2.0055, line width Δ*B*_pp_ = 0.38 mT). Figure 4a shows the dependence of field intensity on the distance between the EPRoC and the permanent magnet. In Figure 4b the broadening (in ppm) of the EPR line width induced by the inhomogeneity of the magnetic field for each position of the permanent magnet with respect to the EPRoC is shown. Spectral simulations and the least-squares fitting routine employed to characterize the obtained data are reported in the Material and Methods section. The distance between the minima of each FM-detected signal was determined from the fit and the broadening of the EPR line width in ppm was calculated using eq 2 and plotted in Figure 4b. The errors were determined from the errors provided by the fitting routine of the esfit function.^47^ An example of FM-detected signal of the a-Si sample can be found in the “a-Si FM-detected EPR signal” section in the Supporting Information.

### Spin Counting and Molecular Tumbling with EPRoC

**Figure 5:**
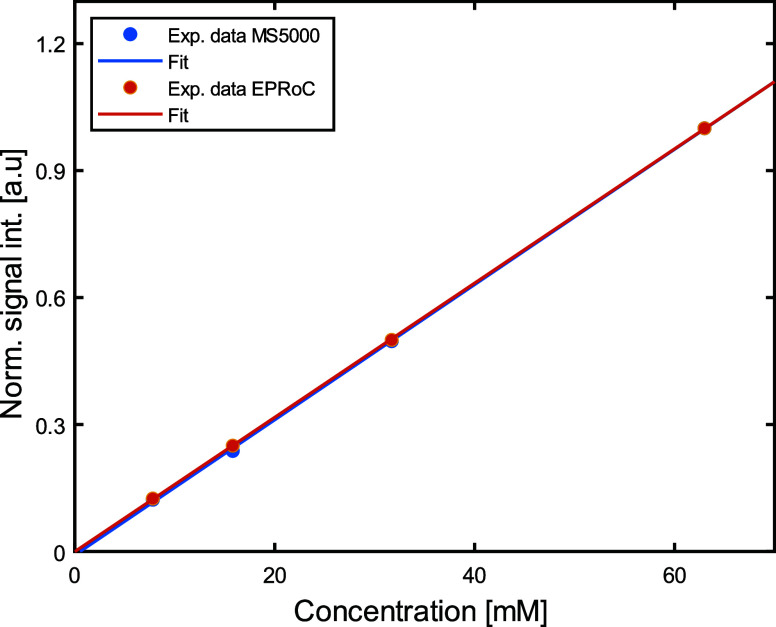
EPR signal intensity obtained by second integration of the data measured using the MS5000 (blue dots) and using the EPRoC with the permanent (orange dots) plotted as a function of tempol concentration. The data have been normalized to the maximum value obtained for the highest sample concentration measured (63 mM). The solid lines show the best fit to the data assuming a linear relation. Error bars are within the size of the data points.

The information about the homogeneity and the intensity of *B*_0_ obtained after the mapping procedure was used to calibrate the position of the EPRoC with respect to the permanent magnet in the region of highest homogeneity to perform proof of principle EPR experiments. Tempol solutions of different concentrations (63, 31.7, 15.8, and 7.9 mM) dissolved in a solution of 20% water and 80% glycerol were measured to establish a calibration procedure for quantitative measurements using the benchtop Magnettech MS5000 spectrometer and the EPRoC in combination with the permanent magnet. For these measurements, the distance between the EPRoC and the permanent magnet was kept constant at 300 μm. The signal intensity of the data measured with the Magnettech MS5000 spectrometer was obtained by numerical double integration of each recorded spectrum while polynomial baseline correction was performed before each integration. Since the spectra measured with EPRoC are dispersion-like signals, the FM-signals were Hilbert transformed to absorption-like signals using the Kramers–Kronig relations.^50^ It should be noted that Kramers–Kronig relations can be applied only for signals that are not power saturated.^50^ Accordingly, the measurements with the EPRoC have been performed for *B*_1_ = 20 μT which was confirmed to still be in the linear, unsaturated regime as determined from the power–saturation curve of the tempol solutions using the Magnettech MS5000. Subsequently, the same double integration and baseline correction procedures were performed to obtain the signal intensity. The results of the measurements are shown in Figure 5. The signal intensity normalized to the maximum value obtained for the highest concentration is plotted as a function of the sample concentration. A linear fit has been performed for each data set and good agreement was found between the angular coefficients *m*_MS500_ = 0.0159(3) and *m*_EPRoC_ = 0.0158(2), for the MS5000 and EPRoC, respectively.

**Figure 6:**
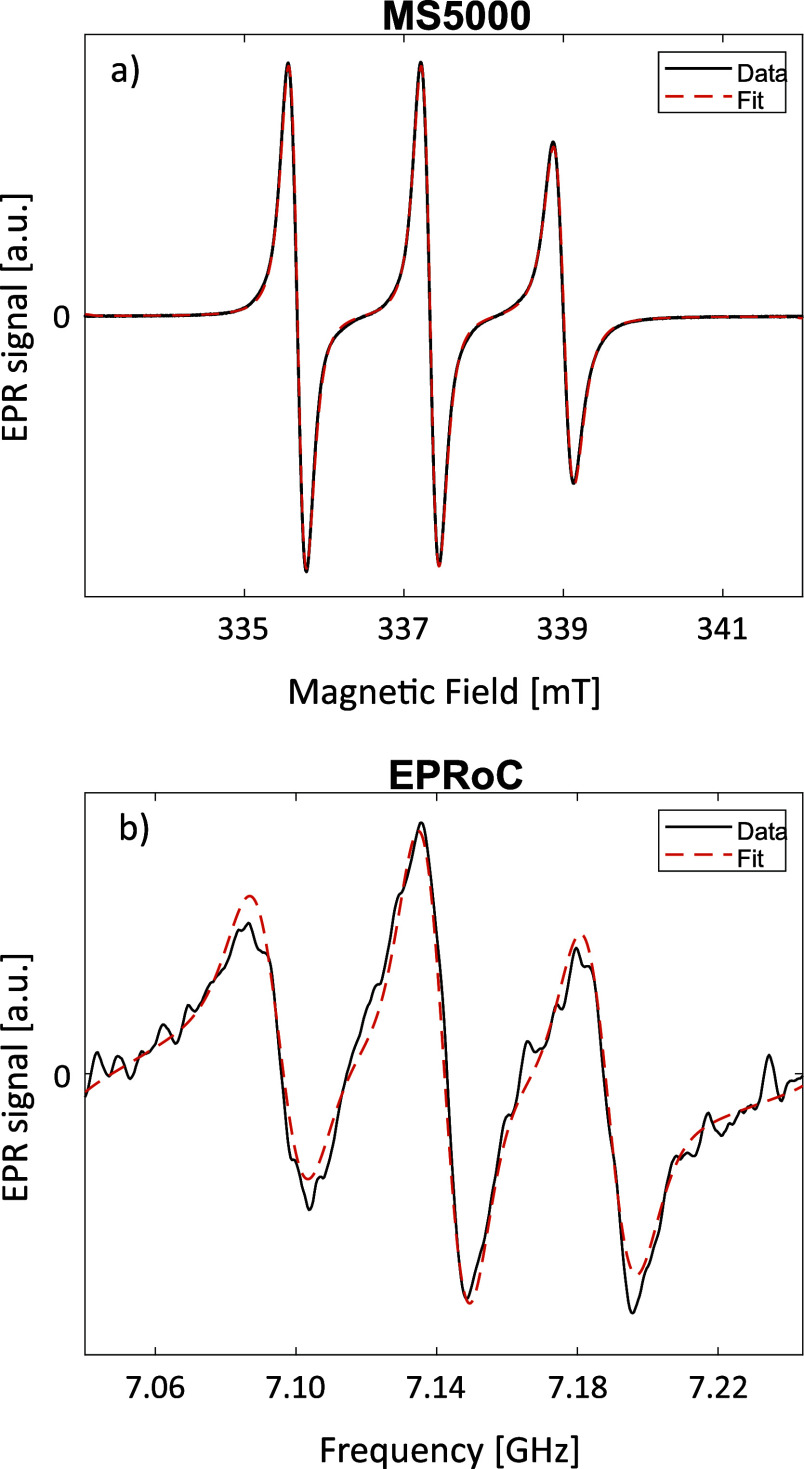
(a) EPR signal of 31.7 mM tempol dissolved in 80% glycerol and 20% water. The black trace represents the experimental data measured using the benchtop EPR spectrometer (Magnettech MS5000) and the red trace is the result of the fit to the data. (b) EPR signal of 31.7 mM tempol dissolved in 80% glycerol and 20% water measured using the EPRoC and permanent magnet. The absorption signal has been obtained by Hilbert transformation of the FM-detected signal via Kramers–Kronig relations. The black and red traces represent the experimental data and the fit, respectively. The spectrum has been filtered using a 2nd order Savitzky–Golay filter with a window chosen to be small enough to ensure a line broadening <5%. Since the same sample was measured using the two different EPR experimental configurations, the line width discrepancy may therefore be attributed to the inhomogeneity of the magnetic field of the permanent magnet.

After determining the linear dependence of the EPR signal on concentration, the spectra of the 31.7 mM tempol dissolved in 80% glycerol and 20% water solution and placed in the flat capillaries were analyzed in further detail. The typical three-line pattern resulting from the hyperfine interaction of the electron spin with the nitrogen nuclear spin,^51^ measured using both the Magnettech MS5000 and the EPRoC and permanent magnet, is shown in Figure 6. It should be noted that the sample was used when measuring with both spectrometers to facilitate comparative data evaluation without consideration of sample environment variations. The EPRoC FM-detected signal was measured by sweeping the frequency of the EPRoC, and subsequently Hilbert transformed to an absorption signal. The data were fitted using the esfit function provided by the EasySpin^47^ library employing the chili function for the slow-motion regime, due to the high viscosity of glycerol as explained in the Material and Methods section. For the simulation, a rhombic *g* factor (*g* = [2.0092 2.0061 2.0059]) and hyperfine tensor (*A* = [0.55 0.63 0.359] mT) were used, and are consistent with literature reports.^52,53^ The line width (Δ*B*_pp_) and tumbling correlation time (τ_R_) obtained from the fit were compared to results in the literature (Table 1).

**Table 1:** Results of the Fitting Procedure Performed on the Data Measured Using the Magnettech MS5000 and the EPRoC for the Line Width (Δ*B*_pp_) and the Tumbling Correlation Time (τ_*R*_) of the 31.7 mM Tempol in 80% Glycerol and 20% Water Solution. Values from the Literature are Shown for Comparison

	MS5000	EPRoC	literature^38^
Δ*B*_pp_	0.19(3) mT	0.59(4) mT	0.16 mT
τ_R_	0.34(2) ns	0.56(5) ns	0.39 ns

## Discussion

The characterization of the permanent magnet, presented in the previous sections, demonstrates the applicability of the EPRoC as a sensor for imaging static magnetic fields. The 2D maps of the magnetic field intensity and the signal broadening shown in Figure 3c,d, respectively were obtained with a spatial resolution of 100 μm × 50 μm along the *x*- and *y*-directions in a range of 1.2 mm × 5 mm. The spatial resolution was mainly determined by the size of the BDPA grain (≈200 μm diameter) placed on top of one array detector. Hence, higher spatial resolution in the micrometer range may be obtained using smaller samples. The results in Figure 3c allow for the calculation of the average value of  over the investigated region of the permanent magnet. In Figure 3d the broadening obtained from the observed BDPA line width (eq 2) is shown. In the region of highest homogeneity, the line width broadening is ≈700 ppm. This region extends for ≈5 mm along the *x*-direction and ≈0.6 mm along the *y*-direction. Since the diameter of each coil of the VCO is 200 μm, it is possible to place the whole 2 × 7 array in this region with highest homogeneity in order to utilize all 14 coils for EPR measurements (Figure 3d). The results in ppm obtained using eq 2 are largely dependent on the choice of the paramagnetic probe (BDPA). For probes that exhibit very small line widths, large line broadening values are obtained. Conversely, for probes whose line width is greater than the magnetic field inhomogeneity, the line broadening value tends to zero. Therefore, to characterize homogeneous magnetic fields, it is necessary to select samples with a very small line width. This approach allows for direct determination of the quality of magnets used in EPRoC spectrometers and to evaluate their potential for EPR spectroscopy applications. It should be noted that when characterizing more homogeneous permanent magnets using samples with narrow line widths, it is essential to ensure that the magnetic field homogeneity of the electromagnet is high enough to prevent line width broadening of the reference sample. The height dependence of the magnetic field was determined with an a-Si sample covering the whole VCO array. The results in Figure 4a show that the field strength increases along the vertical direction perpendicular to the *x*–*y* plane of the permanent magnet. However, the signal broadening (Figure 4b) up to a distance of ≈600 μm from the magnet surface is ≈150 ppm. Thus, the homogeneity of the permanent magnet is sufficient to measure samples with line widths slightly larger than 0.4 mT with the magnet used here without introducing signal broadening. These results, combined with the mapping procedure performed using the BDPA sample, demonstrate the possibility to perform 3D magnetic field imaging using either a single coil or all 14 coils of the VCO array. In this way, the region of best homogeneity of the magnetic field can be accessed to perform EPR measurements. The EPRoC and the permanent magnet have been used to measure tempol with different spin concentrations (63, 31.7, 15.8, and 7.9 mM) dissolved in 80% glycerol and 20% water solutions to establish a calibration procedure for spin counting. The findings shown in Figure 5 indicate a clear linear relationship between the signal intensity acquired using the benchtop MS5000 spectrometer and the EPRoC in combination with the permanent magnet, thereby demonstrating the viability of quantifying the number of spins in other samples, when placed in a flat capillary or other uniform sample geometry, by computing the signal intensity. The 31.7 mM tempol solution was then analyzed in further detail to evaluate the possibility of determining the tumbling correlation time of the tempol molecules. As it has been already demonstrated in other works,^38^ the high viscosity of glycerol decreases the tumbling rate of the nitroxide and this effect results in different intensities of the three spectral lines. This effect is well depicted in the differing peak intensities observed for the measurements shown in Figure 6a,b, and it is further described by the results of the fit reported in Table 1. The values of the line width Δ*B*_pp_ = 0.19(3) mT and correlation time τ_c_ = 0.34(2) ns measured with the benchtop MS5000 spectrometer are in good agreement with the findings in ref (38). The tumbling correlation time measured with the EPRoC is about a factor of 1.6 larger than the value obtained with the MS5000. This discrepancy is due to the signal broadening introduced by the inhomogeneity of the magnetic field of the permanent magnet. Indeed, the line width Δ*B*_pp_ measured with the EPRoC is about a factor of 3 larger than the value obtained with the MS5000. As described in,^54^ the line width variation due to broadening effects is particularly relevant for correlation time τ_c_ < 1 ns. Despite the discrepancies in the results, the EPRoC and the permanent magnet used in this work exhibit potential for accurate measurements of the tumbling correlation time of spin label nitroxides in the slow-motion regime (τ_c_ > 1 ns) due to the greater value of line widths (typically Δ*B*_pp_ > 0.4 mT).^51^

## Conclusions and Outlook

We have successfully demonstrated a portable EPR spectrometer based on a small single-sided permanent magnet, combined with a 7 GHz array EPRoC. The EPRoC sensor was used to determine the spatial distribution of the strength and homogeneity of the magnetic field *B*_0_ of the permanent magnet, which is shown to be well-suited for measuring undistorted spectra with a line width larger than 0.4 mT. The combined system was used to determine the molecular tumbling correlation time of tempol in glycerol, showing a discrepancy of a factor 1.6 with respect to measurements with a conventional EPR spectrometer and to what has previously been reported in the literature. This is attributed to line width broadening induced by the inhomogeneity of the magnetic field. However, it was possible to demonstrate that the broadening of the EPR line width does not represent a limiting factor for the quantitative determination of spin concentration, the inhomogeneous line broadening would only reduce the signal-to-noise ratio (S/N). To realize higher magnetic field homogeneity in future, specifically with respect to S/N in case of narrow lines, the design can be further refined by using an ablation laser to directly inscribe the corrective pattern onto the surface of the magnet segments. This avoids the use of the μ-strip, which is hard to be accurately align and making the mechanical assembly and the lasing process more precise and less susceptible to errors. Overall, the EPRoC represents a robust solution for magnetic field imaging application.^26^ The EPRoC used in this work has a bandwidth of ≈1 GHz, which allows the mapping of magnetic fields over a range of 34 mT. New designs of the EPRoC device with a larger bandwidth would allow mapping of the magnetic field in a much broader field intensity range. Moreover, we have demonstrated the applicability of the EPRoC device and the permanent magnet for spin counting applications, and for molecular tumbling determination of nitroxides in the slow-motion regime. Further improvements of the permanent magnet homogeneity will ensure precise determinations of the molecular correlation time in the fast-motion regime and at the isotropic limit as well.^51,54^ Moreover, the compact size and single-sided design of the permanent magnet allow, in combination with the EPRoC, the develop of a dipstick EPR sensor which can be immersed directly into the sample environment.^55^ Overall, the EPRoC and the permanent magnet represent a promising solution for the development of a new generation of versatile and compact EPR spectrometers, which could be driven by a battery for in situ, in vivo and operando spectroscopic applications in the field of energy materials, healthcare, food quality control and catalysis to determine surface spin concentration with great potential for industrial applications.

## Supporting Information Available

B_0_ mapping. A derivation of the broadening parameter and the recorded FM spectrum of the a-Si sample are included (PDF)
