# Supplementary material for: Compact Electron Paramagnetic Resonance on a Chip Spectrometer Using a Single Sided Permanent Magnet
Source: ACS Sens. 2024 Sep 26;9(10):5099–108. doi: 10.1021/acssensors.4c00788 (PMC11519922; doi:10.1021/acssensors.4c00788)
Supplement: Supplementary file 1 — se4c00788_si_001.pdf [file se4c00788_si_001.pdf]

Supporting information

Compact Electron Paramagnetic Resonance on a Chip spectrometer using a single sided permanent magnet.

Michele Segantini,[1] Gianluca Marcozzi,[1] Tarek Elrifai,[2] Ekaterina Shabratova,[1,3] Katja Höflich,[3] Mihaela Deaconeasa,[4] Volker Niemann,[4] Rainer Pietig,[4] Joseph E. McPeak,*[1] Jens Anders,[2,5] Boris Naydenov,[1] Klaus Lips[1,6]

[1]Helmholtz-Zentrum Berlin für Materialien und Energie GmbH, Hahn-Meitner-Platz 1, 14109 Berlin, Germany

[2]Institute of Smart Sensors, Universität Stuttgart, 70569 Stuttgart, Germany

[3]Ferdinand-Braun-Institut gGmbH (FBH), Leibniz-Institut für Höchstfrequenztechnik 12489 Berlin, Germany

[4]Bruker BioSpin GmbH, 76275 Ettlingen, Germany

[5]Center for Integrated Quantum Science and Technology (IQST), 70569 Stuttgart and Ulm, Germany

[6]Berlin Joint EPR Laboratory, Fachbereich Physik, Freie Universität Berlin, 14195 Berlin, Germany

Broadening derivation

In this appendix, we will explain the derivation of Eq. 2 used to determine the EPR signal broadening induced by the inhomogeneity of the permanent magnet. In Fig. S1, two simulated EPR absorption spectra are shown where the two spectra have been simulated using the *pepper* function from EasySpin[1] with a value of the g-factor of the free electron ($g_e = 2.0023$) and arbitrary peak-to-peak linewidth respectively $\Delta B_{pp} = 8.4$ MHz (blue curve) and $\Delta B_{pp} = 15.4$ MHz (orange curve). The linewidth peak-to-peak of the two curves is defined as follow:

$$L = f_2 - \mathrm{f}_1 \quad \text{Eq. A1}$$

$$L' = f'_2 - f'_1 \quad \text{Eq. A2}$$

Assuming that the two lines are correlated by some broadening effect, the maxima and minima relate to the following relations:

$$f'_1 = f_1 - \Delta \mathrm{f}_1 \quad \text{Eq. A3}$$

$$f'_2 = f_2 + \Delta \mathrm{f}_2 \quad \text{Eq. A4}$$

where $\Delta \mathrm{f}_1$ and $\Delta \mathrm{f}_2$ represent the increments along the frequency axis of the peaks due to the broadening. Thus, it is possible to express L' as a function of L using the Eq. A3 and Eq. A4:

$$L' = L + \Delta \mathrm{f}_1 + \Delta \mathrm{f}_2 \quad \text{Eq. A5}$$

It is thereby possible to express Δf_1 and Δf_2 as follow:

$$\Delta f_1 = f_1 \cdot \Delta B_{ppm} \qquad \text{Eq. A6}$$

$$\Delta f_2 = f_2 \cdot \Delta B_{ppm} \qquad \text{Eq. A7}$$

where ΔB_{ppm} is the signal broadening in ppm. Combining the Eq. A6, Eq. A7 and Eq. A5 we obtain Eq.2

$$\Delta B_{ppm} = \frac{L' - L}{f_1 + f_2}\ . \qquad \text{Eq. 1}$$

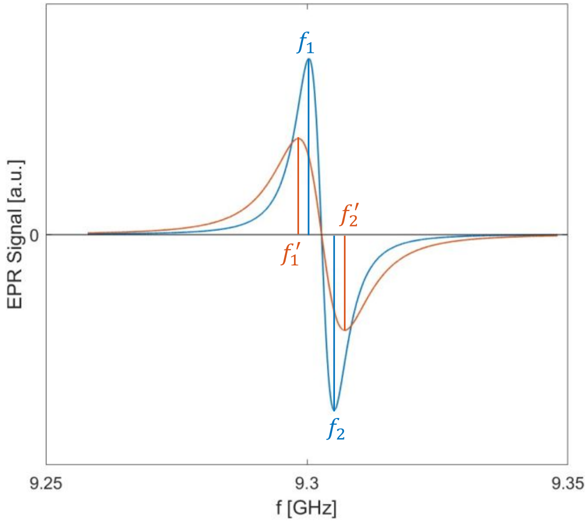

Figure S1 Simulation of two absorption EPR spectra using the pepper function from EasySpin. The simulations have been performed using the g-factor of the free electron and linewidth $\mathbf{\Delta B_{pp} = 8.4\ MHz}$ for the blue curve and $\mathbf{\Delta B_{pp} = 15.4\ MHz}$. The points $\boldsymbol{f_1}$ and $\boldsymbol{f'_1}$ correspond to the maxima of the two curves, and $\boldsymbol{f_2}$ and $\boldsymbol{f'_2}$ to the minima. The parameters of the simulation have been selected in such way to explain the concept behind the derivation of the Eq. 1.

a-Si FM-detected EPR signal

The FM-detected EPR signal of the a-Si thin film sample measured using the EPRoC and the permanent magnet are shown in Fig. S2. The experimental settings and parameters used to perform these measurements are described in detail in the Material and Methods section of the main text. The data were fitted using the *esfit* function provided by EasySpin[1] library employing the *pepper* function. For the simulation, a rhombic g factor (g=[2.0079 2.0061 2.0034]), g-strain = [0.0054 0.0022 0.0018] and hyperfine tensor (A = [151 151 269]) were used are consistent with literature values[2]. The linewidth ΔB_{pp}= 14.3(4) MHz was obtained from the fitting procedure.

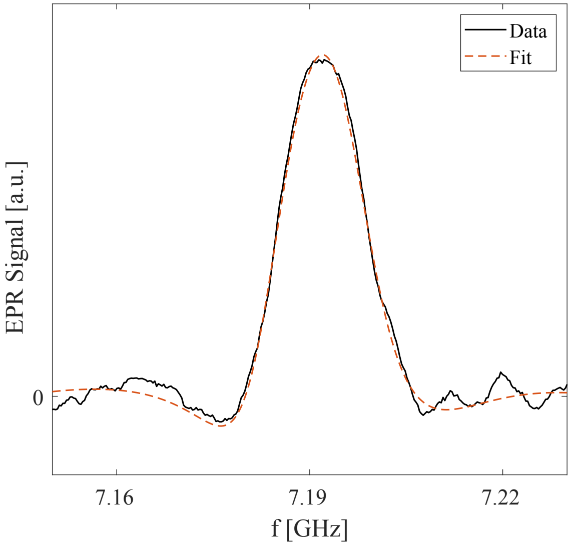

Figure S2 The FM-detected EPR signal of the thin film a-Si sample. The black trace represents the experimental data measured using the EPRoC and the permanent magnet. The red trace is the result of the fitting procedure performed using the *esfit* function of the EasySpin library. The spectrum was filtered using a 2nd order Savitzky-Golay filter with a window chosen to ensure a linewidth broadening < 5%.

REFERENCES

(1) Stoll, S.; Schweiger, A. EasySpin, a Comprehensive Software Package for Spectral Simulation and Analysis in EPR. *Journal of Magnetic Resonance* **2006**, *178* (1), 42–55. https://doi.org/10.1016/j.jmr.2005.08.013.

(2) Fehr, M.; Schnegg, A.; Rech, B.; Lips, K.; Astakhov, O.; Finger, F.; Freysoldt, C.; Bittl, R.; Teutloff, C. Dangling Bonds in Amorphous Silicon Investigated by Multifrequency EPR. *Journal of Non-Crystalline Solids* **2012**, *358* (17), 2067–2070. https://doi.org/10.1016/j.jnoncrysol.2011.12.105.
